# Low-dose GBCA administration for brain tumour dynamic contrast enhanced MRI: a feasibility study

**DOI:** 10.1038/s41598-024-53871-x

**Published:** 2024-02-28

**Authors:** Daniel Lewis, Ka-Loh Li, Mueez Waqar, David J. Coope, Omar N. Pathmanaban, Andrew T. King, Ibrahim Djoukhadar, Sha Zhao, Timothy F. Cootes, Alan Jackson, Xiaoping Zhu

**Affiliations:** 1 Department of Neurosurgery, Manchester Centre for Clinical Neurosciences, Salford Royal NHS Foundation Trust, Manchester Academic Health Science Centre, Manchester, UK; 2 Geoffrey Jefferson Brain Research Centre, University of Manchester, Manchester, UK; 3 Division of Neuroscience and Experimental Psychology, School of Biological Sciences, Faculty of Biology, Medicine and Health, University of Manchester, Manchester, UK; 4 Division of Informatics, Imaging and Data Sciences, School of Health Sciences, Faculty of Biology, Medicine and Health, University of Manchester, Manchester, UK; 5 Division of Cancer Sciences, School of Medical Sciences, Faculty of Biology Medicine and Health, University of Manchester, Manchester, UK; 6 Division of Cell Matrix Biology and Regenerative Medicine, School of Biological Sciences, Faculty of Biology Medicine and Health, University of Manchester, Manchester, UK; 7 Division of Cardiovascular Sciences, School of Medical Sciences, Faculty of Biology Medicine and Health, University of Manchester, Manchester, UK; 8 Department of Neuroradiology, Manchester Centre for Clinical Neurosciences, Salford Royal NHS Foundation Trust, Manchester Academic Health Science Centre, Manchester, UK; 9 Department of Neurosurgery, Manchester Centre for Clinical Neurosciences, Salford Royal NHS Foundation Trust, Stott Lane, Salford, Greater Manchester M6 8HD UK

**Keywords:** Diagnostic markers, CNS cancer, Neurology, Brain, Magnetic resonance imaging, Brain imaging

## Abstract

A key limitation of current dynamic contrast enhanced (DCE) MRI techniques is the requirement for full-dose gadolinium-based contrast agent (GBCA) administration. The purpose of this feasibility study was to develop and assess a new low GBCA dose protocol for deriving high-spatial resolution kinetic parameters from brain DCE-MRI. Nineteen patients with intracranial skull base tumours were prospectively imaged at 1.5 T using a single-injection, fixed-volume low GBCA dose, dual temporal resolution interleaved DCE-MRI acquisition. The accuracy of kinetic parameters (v_e,_ K^trans^, v_p_) derived using this new low GBCA dose technique was evaluated through both Monte-Carlo simulations (mean percent deviation, PD, of measured from true values) and an in vivo study incorporating comparison with a conventional full-dose GBCA protocol and correlation with histopathological data. The mean PD of data from the interleaved high-temporal-high-spatial resolution approach outperformed use of high-spatial, low temporal resolution datasets alone (p < 0.0001, t-test). Kinetic parameters derived using the low-dose interleaved protocol correlated significantly with parameters derived from a full-dose acquisition (p < 0.001) and demonstrated a significant association with tissue markers of microvessel density (p < 0.05). Our results suggest accurate high-spatial resolution kinetic parameter mapping is feasible with significantly reduced GBCA dose.

## Introduction

Dynamic contrast enhanced magnetic resonance imaging (DCE-MRI) is a contrast-based imaging technique for interrogating tumour microenvironment and microvasculature. In brain tumours, studies have shown that microvascular kinetic parameters derived from DCE-MRI can be used not only as predictive and prognostic growth biomarkers, but also for interrogating treatment response in patients undergoing anti-angiogenic treatment or radiotherapy^1–5^.

A key limitation in current DCE-MRI techniques, however, is the requirement during data acquisition for full-dose contrast agent administration (0.1 mmol/kg)^6,7^. Gadolinium-based contrast agents (GBCAs) are widely used in research and clinical settings and each year over 30 million doses of GBCA are administered worldwide^7,8^. An emerging concern, however, is the clinical and experimental evidence demonstrating brain gadolinium deposition in patients undergoing repeated GBCA exposures, with a demonstrated association between serial GBCA administrations and T1 hyperintensity in brain areas such as the dentate nucleus and globus pallidus^6,7,9^. Environmental sustainability concerns have even been raised with growing evidence of gadolinium contamination of natural water systems in the vicinity of large imaging centers^10^.

Although most pre-clinical and clinical studies undertaken to date have not shown evidence of either histological change or clinical sequelae related to gadolinium deposition in the brain^6,7,9,11^, further data from large patient studies on the biodistribution, pharmacokinetics and long-term effects of GBCA exposure is needed. Steps to limit the GBCA dose given during contrast examinations such as DCE-MRI should therefore be undertaken. Beyond long-term safety concerns, use of lower GBCA doses also reduces confounding water exchange^12–14^ and T2^*^ effects^15^, thereby permitting both more accurate measurements of GBCA concentration in tissues of interest^16^ and better vascular input function (VIF) estimation^17,18^. Use of dual temporal resolution (DTR) DCE-MRI acquisitions, which incorporate an initial low-dose, high-temporal resolution (LDHT) pre-bolus for VIF estimation, and a separate standard or full-dose GBCA administration for the tissue residue function can improve subsequent kinetic parameter estimation^18,19^. These LDHT DCE-MRI datasets can also be used directly for low spatial resolution kinetic parameter mapping and estimation of absolute CBF^2–4,18,20^, but a key limitation of these datasets is that the spatial resolution is coarse, which can introduce partial volume errors into VIF estimation and limit assessment of microvascular heterogeneity within small lesions^5,19^.

Recently full GBCA dose dual-injection DCE-MRI (DICE) methods have been developed such as LEGATOS (LEvel and rescale the Gadolinium contrast concentrations curves of high-temporal TO high-spatial DCE-MRI) that address the spatial resolution limitation of high-temporal DCE-MRI and permit derivation of accurate, whole-brain, high-spatial resolution microvascular parameters^5,19^. The requirement for full-dose GBCA administration, however, remained a key limitation of this technique, and the purpose of this study was to determine the feasibility of using a low-dose (3 ml of GBCA) interleaved DCE-MRI protocol with LEGATOS for deriving high-spatial resolution pharmacokinetic parameters. We sought to assess the contrast-to-noise ratio (CNR) and accuracy of derived kinetic parameters using this new low GBCA dose approach through both Monte Carlo simulations and an in vivo study of patients with intracranial tumours with differing enhancement characteristics. We hypothesised that despite the reduced CNR in the low-dose high-spatial-resolution series, this novel approach will generate GBCA enhancement curves with high CNR in the high-temporal arterial phase and low CNR in the high-spatial parenchymal phase, which can be used to generate high-spatial resolution kinetic parameters with better accuracy and precision compared to those obtained from low-dose high-spatial resolution DCE-MRI alone. In addition we hypothesised that within well enhancing tumours use of a low GBCA dose acquisition would provide tissue-validated parameters of comparable accuracy when compared to a full-dose DCE-MRI acquisition.

## Methods

### Patients

For this prospective study nineteen patients with intracranial skull base brain tumours were recruited. The patient group included: three patients with intracranial chordoma involving the clivus; three patients with skull base chondrosarcoma; and thirteen patients with intracranial schwannoma—including one patient with a jugular (IX) schwannoma, eleven patients with sporadic unilateral vestibular schwannoma (VS), and one patient with neurofibromatosis type 2 (NF2) related bilateral VSs. Across all patients there were twenty tumours (14 schwannoma and six chordoma/chondrosarcoma). Schwannomas and chordoma/chondrosarcoma were chosen as disease models for this feasibility study as they display differences in the avidness and heterogeneity of post GBCA enhancement, thereby permitting assessment of this low-dose technique in avidly and poorly enhancing tumours, respectively. Both pathologies are also extra-axial and are therefore less influenced by brain microenvironment and its vasculature. The study obtained ethical approval (NHS Health Research Authority; NRES committee North-west 13/NW/0247) and all patient participants provided informed consent for later analysis of their data. All research was performed in accordance with the Declaration of Helsinki and in alignment with all local policies and guidelines.

### Low-dose DCE-MRI protocol

All patients underwent imaging on a Philips Achieva 1.5 T (Philips Medical Systems) whole body scanner. Patients were imaged using a newly developed, single-injection low GBCA dose DTR DCE-MRI acquisition with an interleaved MRI sampling strategy, comparable to that adopted in previous studies of non-central nervous system (CNS) tumours^21–23^. For all studies, a power injector was adopted and a macrocyclic contrast agent (gadoterate meglumine; Dotarem, Guerbet S.A.) was administered as an intravenous bolus at a rate of 3 ml/s, followed by a chaser of 20 ml of 0.9% saline administered at the same rate.

**Figure 1:**
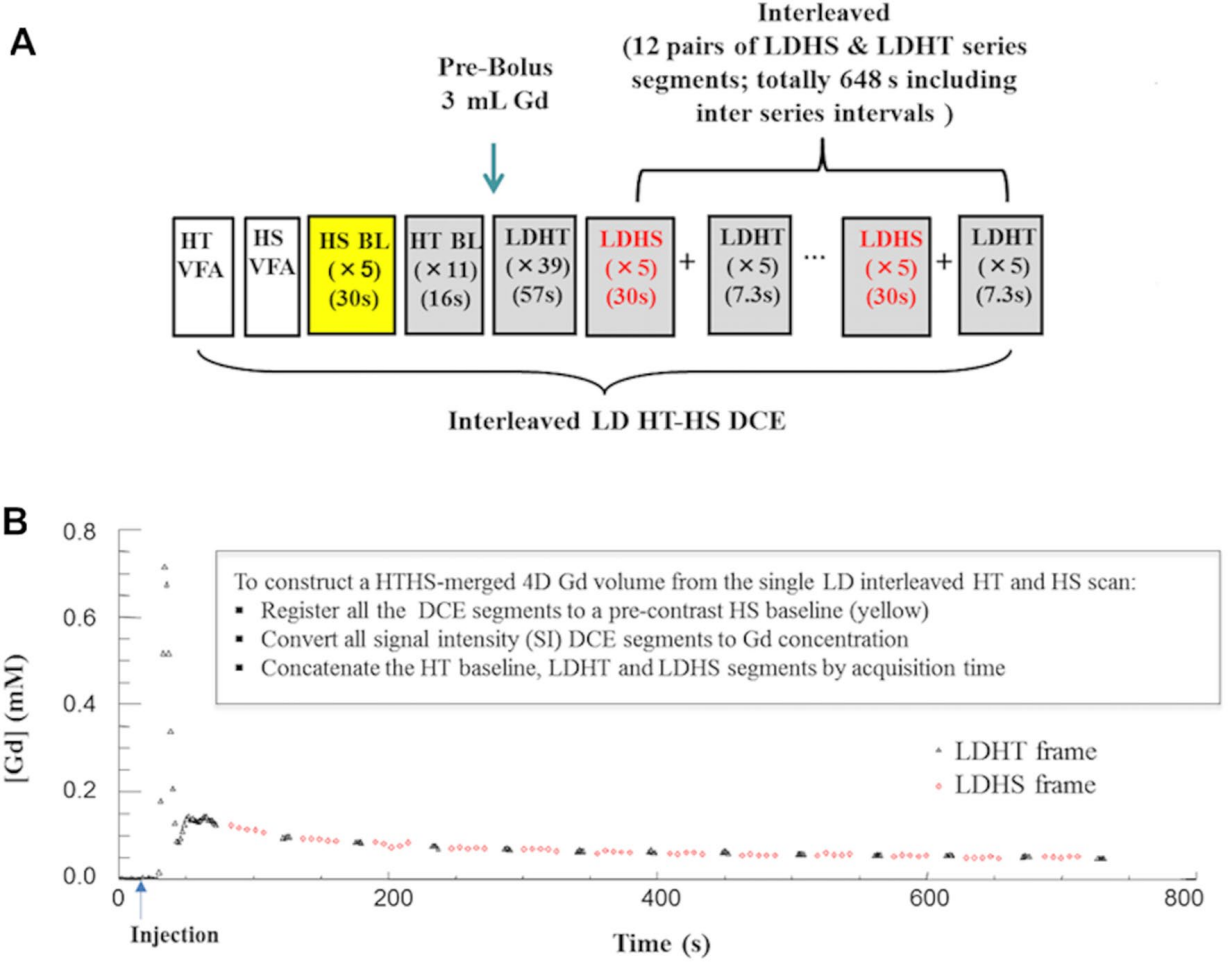
Single-injection, low GBCA dose interleaved DCE-MRI protocol. (**A**) Protocol for the interleaved low GBCA dose high-temporal, high-spatial (HT-HS) interleaved DCE-MRI acquisition. (**B**) Contrast agent concentration–time course measured from a region-of-interest in the superior sagittal sinus. The arterial phase of the concentration–time course is sampled using fifty dynamic HT frames at the start of the acquisition to allow for accurate discrimination between plasma volume and plasma-leakage effects. Later concentration–time points are sampled using twelve pairs of interleaved low-dose LDHS & LDHT series segments. *BL* Baseline frame, *HS* High-spatial, *HT* High-temporal, *LD* Low-dose, *LDHS* Low-dose high-spatial, *LDHT* low-dose high-temporal.

Before and during administration of a low-dose prebolus of contrast agent (3 ml; corresponding to body weight adjusted GBCA dose varying from 0.016 to 0.030 mmol/kg with a mean of 0.020 mmol/kg), an interleaved high-temporal (HT: TR/TE/flip angle of 2.5 ms/0.696 ms/16°; matrix size of 96 × 96 × 28, voxel size of 2.5 × 2.5 × 5 mm^3^, Δt = 1.46 s), and high-spatial (HS: TR/TE/flip angle of 3.7 ms/0.925 ms/16°; matrix size of 240 × 240 × 56, voxel size of 1 × 1 × 2.5 mm^3^, Δt = 6.04 s) resolution acquisition was performed. SENSE (sensitivity encoding) acceleration factor of 1.8, voxel bandwidth of 700 Hz, and a short TR gradient recalled echo-based pulse sequence was used with both gradient spoiler and phase cycling for the elimination of the net transverse magnetization. The gradient spoilers were applied along read (Gx) and slab selection (Gz) directions. Variable flip-angle (α = 2°, 6°, 12° and 16°) acquisitions matched in image dimensions to the HT and HS frames of the interleaved protocol were undertaken for native longitudinal relaxation rate (R1_0_) mapping. An overview of the single low-dose interleaved DTR protocol and time course of the interleaved HT and HS segments are shown in Fig. 1.

For all patients, immediately following the single-injection interleaved DTR series a full dose high-spatial resolution (FDHS; dose = 0.2 mls/kg·weight− 3 ml dose of prebolus) acquisition was also performed. This was to allow for a comparison between kinetic parameters derived using the low-dose interleaved acquisition and a standard full-dose dual-injection protocol. Acquisition parameters for this FDHS acquisition were as follows: TR/TE/flip angle of 3.7 ms/0.93 ms/16°, sense acceleration factor of 2.8, reconstructed matrix size of 240 × 240 × 56, reconstructed voxel size of 1 × 1 × 2.5 mm^3^, voxel bandwidth of 700 Hz and frame duration (Δt) of 6.04 s (n = 70). Zero padding was used for FFT reconstruction in the z-direction, which doubled the number of slices.

Prior to kinetic analysis all images from the low-dose HT (LDHT) and full dose (FDHS) acquisitions were co-registered and resliced to a low-dose HS baseline image frame from the interleaved acquisition. For tumour delineation a high-resolution 3D T1-weighted (T1W) gradient echo (GRE) sequence (TE 3.2 ms, TR 8.6 ms, slice thickness 1.2 mm) was also performed after the FDHS DCE acquisition and therefore following a full GBCA dose.

### DCE-MRI data reconstruction using LEGATOS

Voxelwise maps of the microvascular kinetic parameters K^trans^ (transfer constant), *v*_p_ (fractional plasma volume) and v_e_ (the fractional volume of extravascular extracellular space or EES) were derived using the extended Tofts model (ETM)^24^ and the previously described LEGATOS method^19^.

Errors through temporal jitter uncertainty were first reduced through construction of a merged/concatenated DTR 4D GBCA concentration volume containing a high-temporal (HT) resolution ‘arterial’ phase, followed by a later low temporal, high-spatial (HS) resolution ‘parenchymal’ phase^19,25^. In the second part of this process, the high-temporal but low spatial resolution arterial phase of each voxel concentration curve was then re-scaled using a derived voxelwise calibration ratio (ratio_calib_), to increase the spatial resolution of derived kinetic parameter maps^19^. This voxel-wise calibration method assumes that the observed difference in the GBCA concentration between the concatenated HT and HS concentration curves in each voxel of the merged 4D concentration volume reflects the difference in the native spatial resolution during data acquisition^19^. For each voxel, the calibration ratio was calculated from the concatenated 4D concentration–time curve and defined as: the ratio of the mean concentration of 5 HS frames following the concatenation time point over the mean concentration of four final frames of the HT arterial phase series before the concatenation point. The initial HT arterial phase of each voxel concentration–time curve was then rescaled using this derived calibration ratio so that a smooth concatenation with the later HS parenchymal phase was obtained prior to kinetic fitting.

Two sets of LEGATOS reconstructed maps were generated depending on whether the low-dose HT concentration–time course was concatenated with the low-dose HS series segments (LEGATOS_LDHS;_ total concentration–time course length 751 s) or the separate dedicated full-dose FDHS series segments (LEGATOS_DICE;_ total concentration–time course length 400 s). In all cases the final spatial resolution of derived kinetic parameter maps was 1 × 1 × 2.5 mm. Construction of the DTR 4D concentration volume from the single-injection, low-dose interleaved protocol are illustrated in Fig. 1, and further details on the LEGATOS reconstruction method are provided in prior publications^5,19^.

### VIF extraction and kinetic fitting

For the LEGATOS reconstruction, a combined VIF extracted from the superior sagittal sinus (SSS) is used^19,26^. A previously described semiautomatic extraction method is used to select voxels within the SSS that during the first pass of the GBCA bolus display maximum enhancement^19,20,26^. The VIF was constructed by concatenating all LDHT series segments according to acquisition time (i.e., the initial fifty dynamic HT frames plus the 12 LDHT series segments in the HS-HT interleaved parenchymal phase). These segments are illustrated as the LDHT frames (black triangles) of the GBCA concentration–time curve in Fig. 1B.

For each tissue voxel the GBCA bolus arrival time (BAT) is calculated as part of the fitting and the *C*_p_(t) measured from within the VIF time-shifted to align with the BAT of each tissue voxel contrast agent concentration–time curve^19,26^. A map of scaled fitting error (SFE) was also generated to assess the discrepancy between the original data and the derived curve^19^, using the equation:

$$ {\text{SFE = }}\sqrt {\frac{{\sum\nolimits_{{\text{i}}} {({\text{Ti}} - {\text{Ai)}}^{2} } }}{{\sum\nolimits_{{\text{i}}} {{\text{Ai}}^{2} } }}} $$

where A and T are experimental and theoretical data, respectively. The advantage of using data-based weight in scaling the fitting error is that the resulting SFE has a limited range (0.0–1.0, where SFE = 0 corresponds to the best fitting (T_i_ = A_i_ for all data points), and SFE = 1.0 corresponds to the complete failure in fitting (T_i_ = 0 for all data points)).

### Computer simulations

Monte Carlo simulations were used to investigate the effect on parameter accuracy of potentially low CNR in the high-spatial parenchymal phase of the single low-dose interleaved (HT-HS) DCE-MRI acquisition; and test our hypothesis that this protocol would provide improved accuracy in kinetic parameter estimation when compared with high-spatial but low temporal resolution datasets alone.

The initial step in these simulations involved a comparison of the accuracy and precision of tracer kinetic parameters under three CNR conditions: LDHT, LDHS, and the low-dose HT and HS interleaved DCE-MRI. A simulated tumour tissue concentration–time curve was synthesized using in vivo pharmacokinetic parameter values from an imaged VS (K^trans^ = 0.26 min^−1^; v_p_ = 0.07; v_e_ = 0.5) and converted into SI-time curves. These simulated SI-time curves were then sampled with dual temporal resolution and addition of Rician white noise at three different noise levels, NL = standard deviation (SD)/mean baseline signal: (1) a lower noise level resembling the HT DCE series (mean SI_baseline_ = 371.1; SD SI_baseline_ = 15.2; NL_HT_ = 0.04); (2) a higher noise level resembling the HS DCE series (mean SI_baseline_ = 84.4; SD SI_baseline_ = 12.6; NL_HS_ = 0.15); and (3) a mixed noise level (NL_HT-HS_) resembling the in vivo HT-HS interleaved DCE data (noise-added HT and HS SI-time curves from (1) and (2) were converted back to concentration curves and combined). Kinetic analysis using the ETM was performed with the three different noise levels, and the percent deviations (PD) of the “measured” parameter (K^trans^, v_p_, v_e_) values from the “true” values was calculated as PD = (measured–true)/true × 100. For each noise condition a total of 10,000 repetitions were performed and reported as the mean and SD of PD for each parameter estimate.

Simulations were also used to evaluate the effects of changing ‘true’ tumour K^trans^ on both parameter accuracy and CNR levels within the HS parenchymal tissue phase of the HT-HS derived SI-time curve (CNR_HS parenchymal phase_). Simulations were repeated at different “true” K^trans^ values (K_T_ 0.05, 0.10, 0.15, 0.20, 0.25, 0.30, and 0.35 min^−1^) while keeping the same v_p_, v_e_ and mixed noise level (NL_HT-HS_) as described for the simulations above. The K^trans^ values for these simulations were chosen to reflect the range of mean tumour values seen in previous VS DCE-MRI studies^3–5,19^. We chose to focus on changing K^trans^ rather than v_p_ or v_e_ for this simulation as among the three kinetic parameters estimated by the ETM, K^trans^ has the greatest impact on the enhancement level of the parenchymal tissue phase and is the parameter most reported in clinical trials of anti-vascular agents^1,2,27^. For each simulation, kinetic analysis was performed, and the PD calculated for each estimate. A total of 10,000 repetitions were again performed and reported as the mean and SD of PD for each parameter estimate. For each K^trans^ setting, CNR_HS parenchymal phase_ was calculated as (maximum SI of the LDHS parenchymal tissue phase−mean SI of HS baseline)/SD of HS baseline).

### Tissue analysis

Matched tissue from 10 VSs (nine sporadic and one NF2-related) that had undergone surgical resection were analysed. From each paraffin tissue block serial 5 µm sections were cut and stained with haematoxylin and eosin (H&E) and immunoperoxidase immunohistochemistry (IHC). Tissue sections were assessed for microvessel surface area (CD31) and cell density (H&E) using immunoperoxidase IHC and detailed protocols are described in prior publications^3,4,19^. Ethical approval was obtained for tissue analyses (REC reference 19/NS/0167).

### Statistical analysis

For the computer simulated data, histogram analyses were initially performed to confirm that the distributions of the PD and SFE at the three different noise levels (NL_HT,_ NL_HS_ and NL_HT-HS_) have Gaussian-like distributions (Supplementary Fig. S1**)**. Differences in the percent deviations (PD) for *K*^trans^, *v*_p_, and *v*_e_, and SFE at the three different noise levels were then compared using t-tests. The correlation of different CNR_HS parenchymal phase_ levels (induced through differing ‘true’ tumour K^trans^ settings) with the mean and SD of PD for *K*^trans^ estimates were also reported using Pearson’s product moment correlation coefficient (*r*).

For the in vivo study, paired t-tests and scatterplots were used to compare estimates of mean tumour K^trans^, v_p_ and v_e_ derived using the low-dose LEGATOS_LDHS_ and full-dose LEGATOS_DICE_ methods, respectively. Bland–Altman plots were also used to evaluate the mean of the difference in parameter estimates between the two datasets. For each tumour subgroup, the voxelwise correlation between parameter values obtained using the LEGATOS_LDHS_ and LEGATOS_DICE_ reconstruction methods was compared using Pearson’s product moment correlation coefficient (*r*). The correlation between the full dose LEGATOS_DICE_ derived estimates and the voxelwise difference between LEGATOS_LDHS_ and LEGATOS_DICE_ estimates was also derived for each parameter (v_e_, K^trans^, v_p_) and tumour group. In both cases voxels with an SFE value > 0.5 and voxel values > 99% centile were excluded from the correlation analysis shown.

To assess the effect of low contrast agent dose on LEGATOS-derived parameters and evaluate differences in the CNR between the low-dose LEGATOS_LDHS_ and full-dose LEGATOS_DICE_ approach the SFE of derived parameters using each method was quantified^19,28^. The proportion of fitted voxels with SFE > 0.5 were compared using paired t-tests, and the relationship between the administered contrast agent dose (mmols/kg of body mass) and the proportion of voxels with SFE < 0.5 following the single-injection low-dose interleaved protocol was compared using Pearson’s r.

The inter-tumour correlation between DCE-MRI derived parameter estimates (*K*^trans^, *v*_p_ and *v*_e_) and tissue-derived metrics (H&E cell density and CD31 microvessel surface area) are reported as Pearson’s r or Spearman’s rho in the case of nonlinear associations.

## Results

### Computer simulation

**Figure 2:**
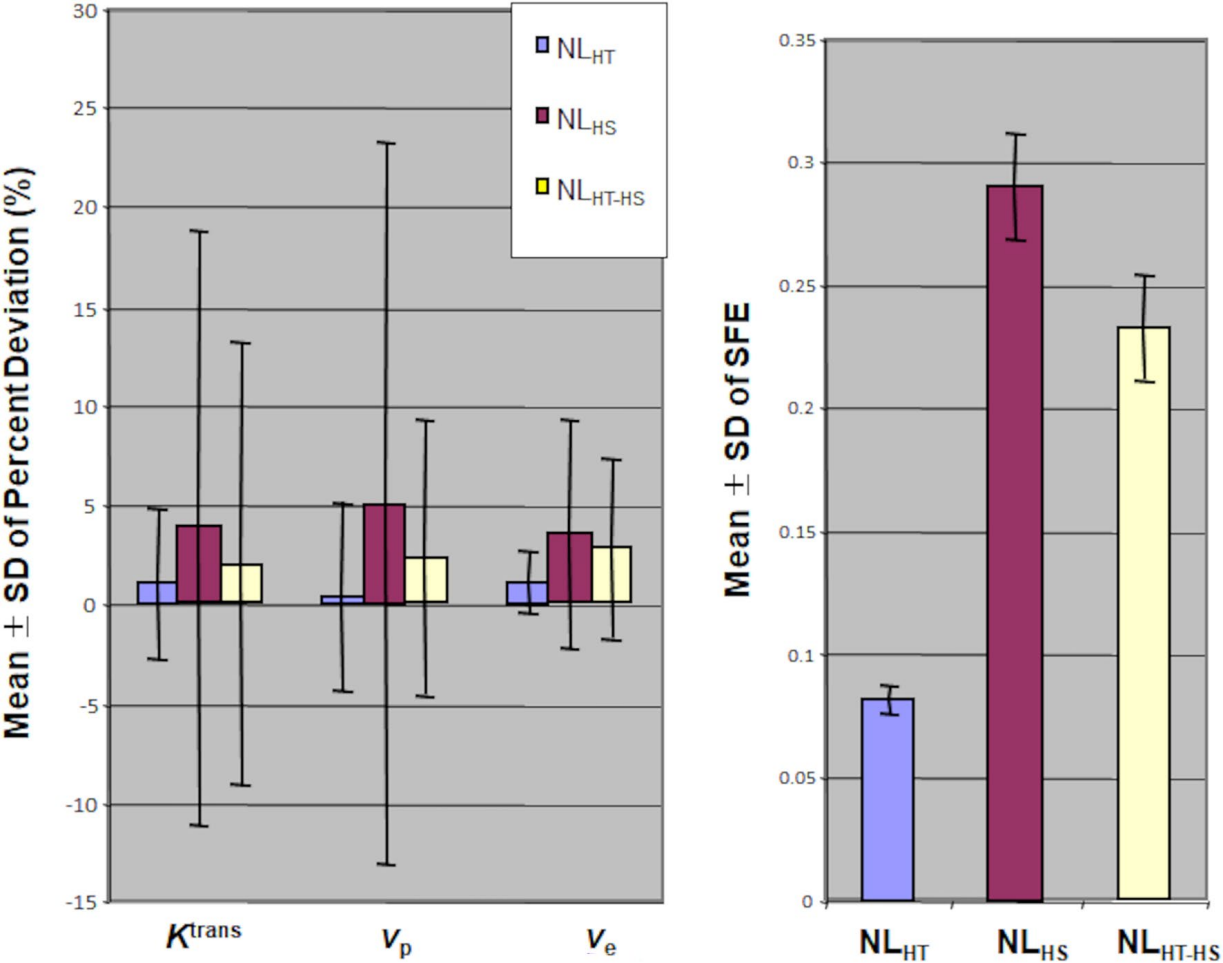
Monte Carlo simulations to compare parameter accuracy at noise levels resembling the in vivo high-temporal (HT) series, the high-spatial (HS) series, and the interleaved HT-HS series when using low GBCA dose. Mean ± SD of percent deviations (PD) for *K*^trans^, *v*_p_, and *v*_e_, and scaled fitting error from 10,000 Monte Carlo repetitions. Simulated tumour tissue concentration–time curve was synthesized using in vivo pharmacokinetic parameter values from an imaged VS tumour with “true” *K*^trans^ = 0.26 min^−1^, *v*_p_ = 0.07, and *v*_e_ = 0.5, and converted into signal intensity (SI)-time curves. Values for three different noise levels (NL) shown: (1) a lower noise level resembling the in vivo HT series, NL_HT_ (= 0.038); (2) a higher noise level resembling the in vivo HS series, NL_HS_ (= 0.12); (3) a mixed noise level, resembling the in vivo HT-HS interleaved series, NL_HT-HS._ Differences in the percent deviations (PD) for *K*^trans^, *v*_p_, and *v*_e_, and SFE between noise levels were compared using paired t-tests.

The effect of low CNR and different noise levels on derived parameter accuracy from the single low-dose HT-HS interleaved DCE-MRI protocol are shown in Fig. 2. Although the mean PD of *K*^trans^, *v*_p_ and *v*_e_ and the mean SFE was higher for the noise level resembling the in vivo HT-HS interleaved SI-time curve (NL_HT-HS_) compared to the lower noise level resembling the HT series (NL_HT_) (P < 0.0001, t-test), it was significantly lower than the mean PD values obtained when using the higher noise level resembling the HS series alone, NL_HS_ (P < 0.0001, t-test).

**Figure 3:**
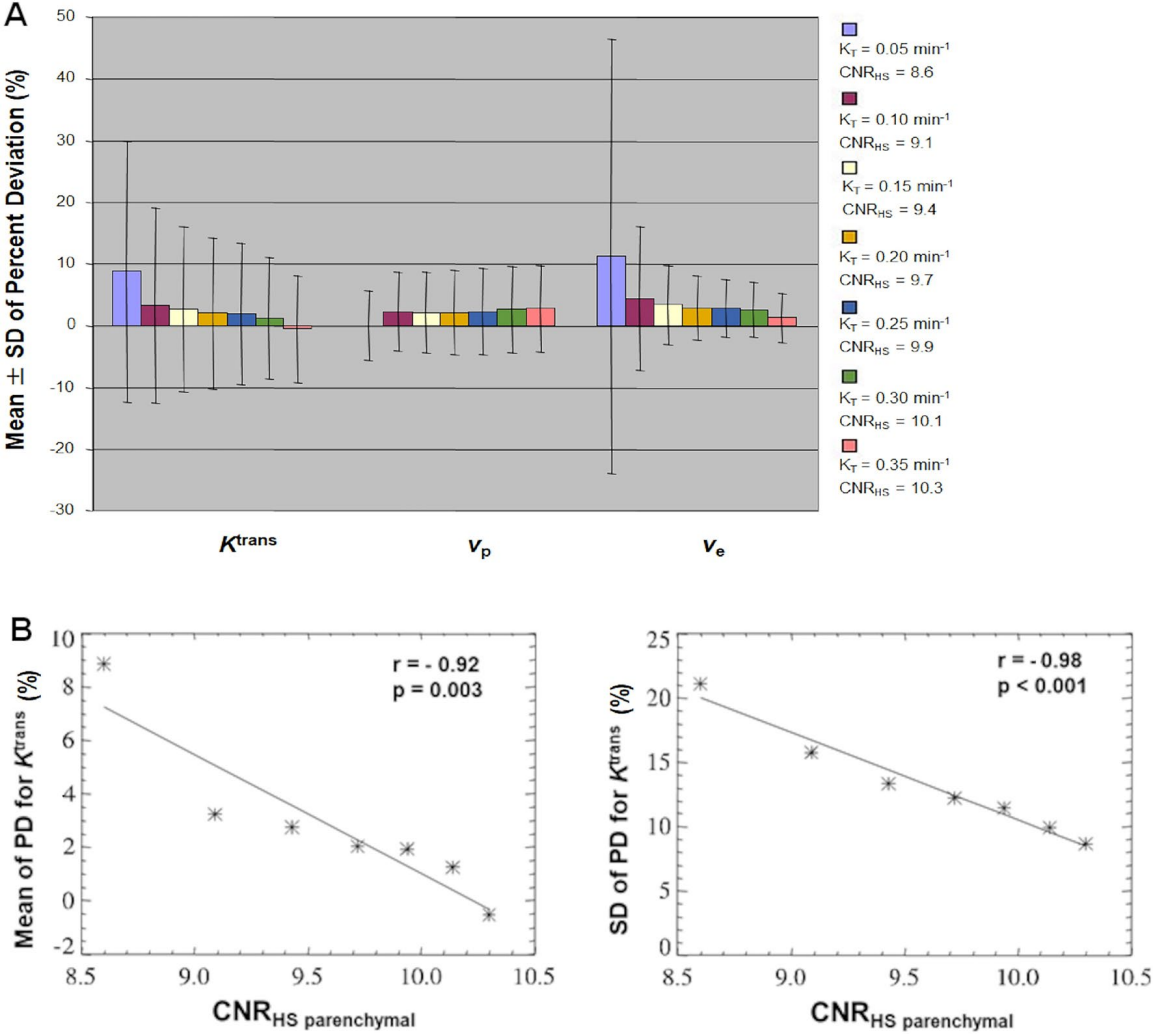
Monte Carlo simulation of the effects of CNR and K^trans^ on parameter accuracy when using interleaved HT-HS acquisition. Simulated low-dose HT-HS interleaved tumour SI-time curve was synthesized using a range of ‘true’ *K*^trans^ values: K_T_ 0.05–0.35 min^−1^; *v*_p_ of 0.07; *v*_e_ of 0.5, and with the mixed noise level (NL_HT-HS_) as used in Fig. 2. (**A**)**:** Mean ± SD of percent deviations (PD) for K^trans^, v_p_, and v_e_ from 10,000 Monte Carlo repetitions at different ‘true’ K^trans^ values (K_T :_0.05–0.35 min^−1^). Increases in ‘true’ K^trans^ from 0.05 to 0.35 min^-1^ resulted in an increase in CNR_HS_ parenchymal phase (8.6 to 10.3) and a corresponding decrease in the mean PD (increased parameter accuracy) of both K^trans^ and v_e_, with minimal effect on the mean PD accuracy of v_p._ (**B**) Scatterplot demonstrating inverse correlation between CNR levels within the HS parenchymal tissue phase of the interleaved HT-HS derived SI-time curve (CNR_HS parenchymal phase_) and the mean (left) or SD (right) percent deviations (PD) in estimated K^trans^ estimates. Correlation reported using Pearson’s product moment correlation coefficient (r). K_T_: true K^trans^; CNR_HS parenchymal phase_: contrast-to-noise ratio of the low-dose high-spatial resolution parenchymal tissue phase. For each K^trans^ setting, CNR_HS-parenchymal phase_ was calculated as (maximum SI of the LDHS parenchymal tissue phase–mean SI of HS baseline)/(SD of HS baseline).

The effect of CNR_HS parenchymal phase_ on parameter accuracy is shown in Fig. 3. Increases in ‘true’ K^trans^ from 0.05 to 0.35 min^−1^ resulted in an increase in CNR_HS parenchymal phase_ (8.6 to 10.3) and a corresponding decrease in the mean PD (increased parameter accuracy) of both K^trans^ and v_e_, with minimal effect on the mean PD accuracy of v_p_ (Fig. 3A). As shown in Fig. 3B there was significant inverse correlation between CNR levels within the HS parenchymal tissue phase of the interleaved HT-HS derived SI-time curve (CNR_HS parenchymal phase_) and the mean (r =− 0.92, p = 0.003) and SD (r =− 0.98, p < 0.001) of percent deviations (PD) in K^trans^ estimates.

### In vivo* evaluation*

Supplementary Fig. S2 shows the tissue-concentration curves ((C_t_(t)) in representative tumour VS voxels from three VS patients injected with 0.030/0.070, 0.020/0.080 and 0.016/0.084 mmol/kg of GBCA when using the low-dose (LDHT-LDHS, *left*) and then full GBCA dose (LDHT-FDHS, *right*) DCE-MRI acquisition, respectively*.* Whereas the first pass of the in vivo acquired C_t_(t) curves are comparable for the two protocols, the parenchymal segment of C_T_(t)^LDHT-LDHS^ are noisier than the C_T_(t)^LDHT-FDHS^, reflecting the lower GBCA dose. Despite the comparatively higher SFE, K^trans^ estimated by fitting typical VS voxel concentration curves from low-dose HT-HS interleaved DCE MRI protocol were comparable to estimates derived from the DICE method, and this was the case across the injected low-dose range of this VS patent cohort, 0.030 (*top*), 0.020 (*middle*), and 0.016 (*bottom*) mmol/kg.

In Supplementary Fig. S3, images of CNR, SFE and LEGATOS derived kinetic parameters from a patient with a sporadic VS imaged using both the low-dose (*top*) and full-dose (*bottom*) acquisition are shown. Despite the GBCA dose difference between these two acquisitions, there was comparable appearance of the CNR and SFE maps within the left sided tumour. Mean K^trans^ estimates from the tumour were also comparable across both with a mean K^trans^ of 0.173 min^−1^ vs 0.175 min^−1^ for the low-dose and full GBCA dose acquisitions, respectively.

**Figure 4:**
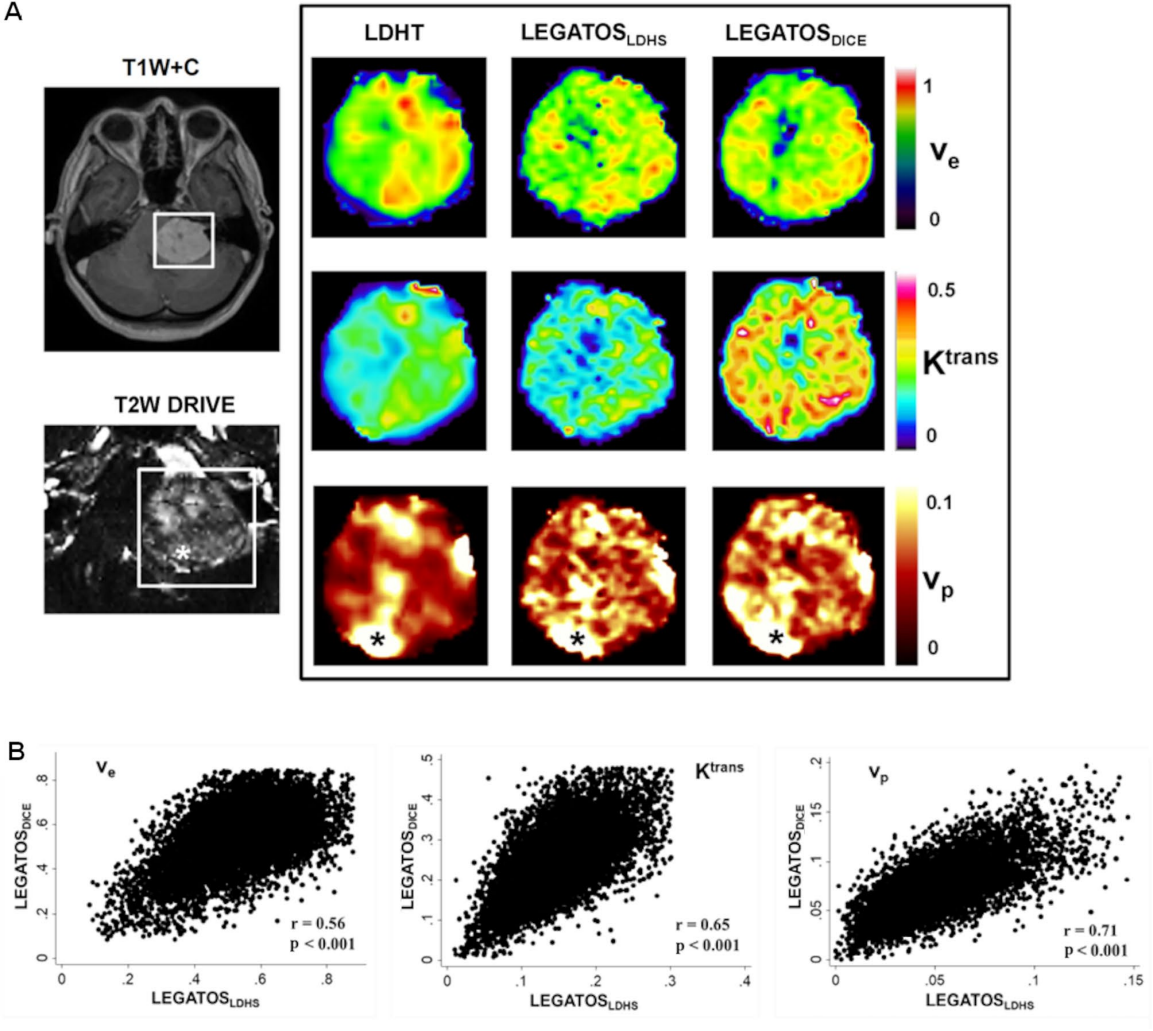
Kinetic parameter maps from a sporadic vestibular schwannoma patient imaged using the single, low GBCA dose interleaved HT-HS DCE-MRI protocol and full GBCA dose (FDHS) acquisition (**A**) Representative images from a patient with a large left sided sporadic VS imaged using the single-injection low-dose interleaved high-temporal and high-spatial resolution (HT-HS) DCE-MRI acquisition followed by a full dose high-spatial resolution (FDHS) acquisition. Kinetic maps derived using either the low-dose LEGATOS_LDHS_ method or the full dose LEGATOS_DICE_ method are shown. Note the increased vascularity around the tumour capsule, visible on both the high-spatial T2W DRIVE acquisition (voxel size = 0.5 × 0.5 × 0.5 × 0.5 mm^3^) and LEGATOS derived *v*_p_ parameter maps ×. (**B**) Scatterplots demonstrating that for all parameters there was a significant voxelwise correlation between the LEGATOS_LDHS_ and LEGATOS_DICE_ derived values (p < 0.001) for the tumour shown in panel A. The overall median voxelwise difference (median % difference) for each of the LEGATOS_LDHS_ derived parameters with respect to LEGATOS_DICE_ estimates was− 0.01 (2.26%),− 0.10 min^−1^ (− 39.2%) and− 0.02 (− 30.0%) for v_e_, K^trans^ and v_p,_ respectively. For all parameters, the difference/bias in voxelwise LEGATOS_LDHS_ estimates increased with increasing voxel values (p < 0.001) with regions of high K^trans^ and v_p_ on the LEGATOS_DICE_ maps showing correspondingly lower LEGATOS_LDHS_ derived parameter values.

In Fig. 4A kinetic parameter maps derived using either the low-dose (LEGATOS_LDHS_) or full-dose (LEGATOS_FDHS_) acquisition are shown for a large, highly vascular sporadic VS. LEGATOS_LDHS_ maps derived from the low-dose interleaved acquisition showed comparable noise to LEGATOS_FDHS_ maps derived from the low-dose HT segments and separate full-dose (FDHS) DCE-MRI data. Although a significant voxelwise correlation between LEGATOS_LDHS_ and LEGATOS_DICE_ parameter values was seen for this tumour (Fig. 4B, p < 0.001), an increase in the difference/bias in voxelwise LEGATOS_LDHS_ estimates with increasing voxel parameter values was observed (p < 0.001), with regions of very high K^trans^ and v_p_ on the LEGATOS_DICE_ maps showing correspondingly lower LEGATOS_LDHS_ derived parameter values, respectively.

**Figure 5:**
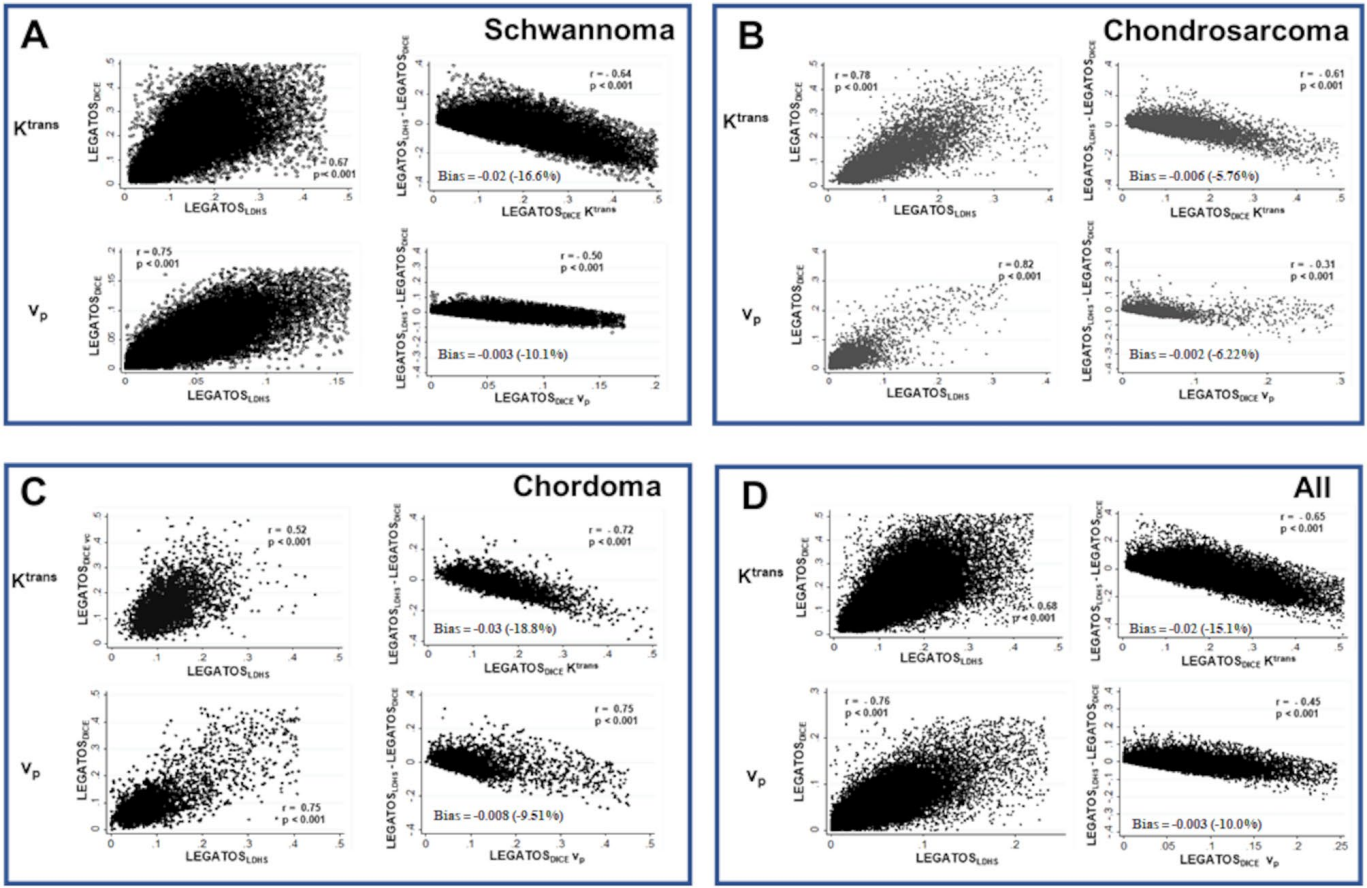
Comparison of voxelwise values of *K*^trans^ and *v*_p_ obtained using the LEGATOS_LDHS_ and LEGATOS_DICE_ reconstruction methods. Values shown for all tumour voxels in the schwannoma (panel A, N = 40,116 voxels), chondrosarcoma (panel B, N = 8079 voxels) and chordoma (panel C, N = 3034 voxels) tumour subgroup along with a pooled analysis of tumour voxels across all three tumour groups (panel D, N = 51,229 voxels). Within each panel the voxelwise correlation between LEGATOS_LDHS_ and LEGATOS_DICE_ derived estimates for K^trans^ (top left) and v_p_ are shown (bottom left). The correlation between the full dose LEGATOS_DICE_ derived estimates and the voxelwise difference between LEGATOS_LDHS_ and LEGATOS_DICE_ estimates is also shown (*top and bottom right*), along with the median bias or voxelwise difference (median % difference relative to LEGATOS_FDHS_ estimate) for each of the LEGATOS_LDHS_ derived parameters. The correlation results are reported using Pearson’s product moment correlation coefficient (*r*). Voxels with an SFE value > 0.5 and voxel values > 99% centile are excluded from the scatterplots shown.

The correlation between LEGATOS_LDHS_ and LEGATOS_DICE_ derived kinetic parameter estimates across all included tumour subtypes are shown in Fig. 5** and **supplementary Fig. S4. Voxelwise analysis demonstrated that for each tumour subgroup, there was a significant voxelwise correlation between parameter values obtained using the LEGATOS_LDHS_ and LEGATOS_DICE_ reconstruction methods respectively (Fig. 5**,** p < 0.001). Across all tumour voxels the median voxelwise difference (median % difference relative to LEGATOS_FDHS_ estimate) for each of the LEGATOS_LDHS_ derived parameters was 0.01 (1.89%),− 0.02 min^−1^ (− 15.1%) and− 0.003 (− 10.0%) for v_e_, K^trans^ and v_p_ respectively, and comparable to the VS shown in Fig. 4 the difference in voxelwise LEGATOS_LDHS_ estimates increased with increasing voxel LEGATOS_DICE_ values (p < 0.001).

Analysis of mean tumour ROI values did not show any statistically significant differences between the mean tumour LEGATOS_LDHS_ and LEGATOS_DICE_ parameter estimates (v_e_, K^trans^, v_p_) in either the schwannoma (n = 14) or chordoma/chondrosarcoma cohort (n = 6) (paired t-test, p > 0.05). The overall correlation between mean tumour *v*_*e*_ estimates was weaker (r = 0.78, p < 0.001) compared to mean tumour *v*_p_ (r = 0.94, p < 0.0001) and *K*^trans^ estimates (r = 0.90, p < 0.001), and corresponding Bland–Altman plots showed the bias/mean difference of each of the LEGATOS_LDHS_ derived parameters with respect to LEGATOS_DICE_ estimates (*v*_e_:− 0.011; *K*^trans^:− 0.015 min^−1^; *v*_p_:− 0.0005**, **Supplementary Fig. S4).

### Comparison between tumour types

There was a non-significant trend to higher v_e_ and K^trans^ values in schwannomas and chondrosarcomas compared to chordomas (Supplementary Table S1). Only in the case of LEGATOS_LDHS_ derived v_e_ estimates though did this difference reach statistical significance (p < 0.05, 1-way ANOVA with Bonferroni correction). For all tumour types the mean SFE was greater for the low-dose LEGATOS_LDHS_ acquisition compared to the full-dose LEGATOS_DICE_ acquisition (p < 0.001, paired t-test). For the imaged schwannoma cohort (N = 14 tumours), for example, the proportion of voxels with SFE > 0.5 was 4.77% (SD: 6.9%) for the dual-injection LDHT-FDHS protocol and 20.1% (SD: 12.9%) of voxels for the single-injection, low-dose interleaved protocol (p = 0.002, paired t-test). There were significant differences in the proportion of voxels with SFE > 0.5 between tumour groups (p < 0.05, 1-way ANOVA with Bonferroni correction) with chordoma and schwannoma showing the highest and lowest SFE, respectively. Despite these differences in SFE, the within group SD in K^trans^ and v_e_ estimates was generally reduced when using the low-dose interleaved protocol (Supplementary Table S1), especially within the VS and chondrosarcoma cohorts, indicating potentially greater parameter estimation precision.

**Figure 6:**
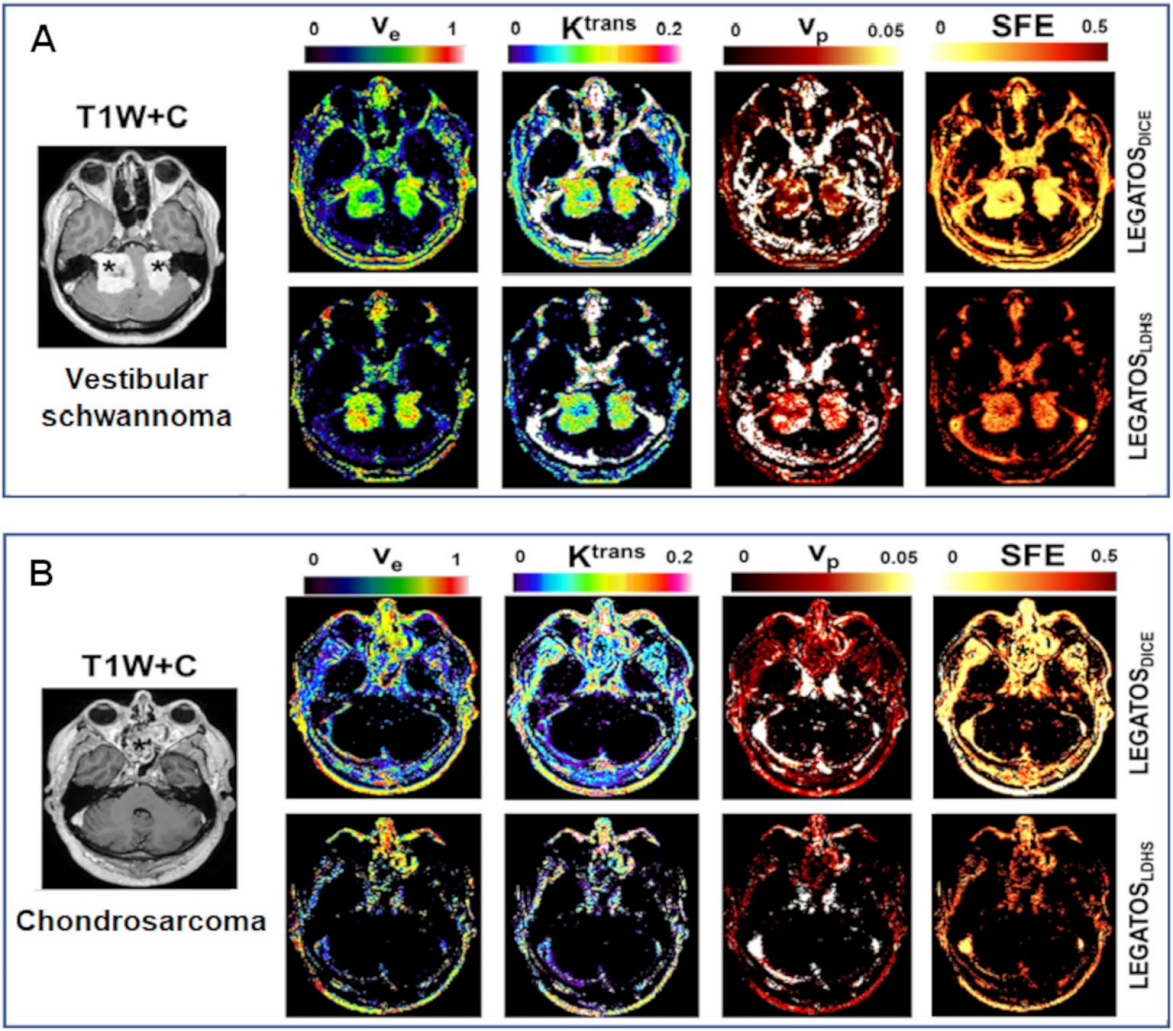
Comparison of fit error and derived kinetic parameters from different skull base tumours. Representative imaging from a patient with bilateral NF2-related VSs (**A**), and a patient with chondrosarcoma involving the anterior cranial fossa (**B**) shown. From left to right: T1W post contrast; parametric v_e_ map; parametric *K*^trans^ map; parametric *v*_p_ map; and map of scaled fitting error (SFE). Kinetic maps and maps of SFE derived using either the low GBCA dose LEGATOS_LDHS_ method or the full GBCA dose LEGATOS_DICE_ method are shown for each patient. Note the more heterogenous enhancement of the skull base chondrosarcoma (*) relative to the VSs, and the much larger difference in SFE within the chondrosarcoma tumour region between the low-dose and full-dose acquisition.

Representative imaging from a patient with bilateral NF2-related VSs and a patient with chondrosarcoma is shown in Fig. 6 and demonstrates the more heterogenous post GBCA enhancement within the imaged chondrosarcoma cohort and the much larger difference in SFE within the chondrosarcoma tumour region between the low-dose and full-dose acquisition. Across all tumours, there was no correlation observed between the administered GBCA dose (mmols/kg of body mass) and the proportion of voxels with SFE > 0.5 following either the low-dose interleaved (LDHT-LDHS) protocol (r =—0.08, p = 0.75) or full-dose (LDHS-FDHS) protocol (r =− 0.25, p = 0.29).

### Imaging and pathology analysis

**Figure 7:**
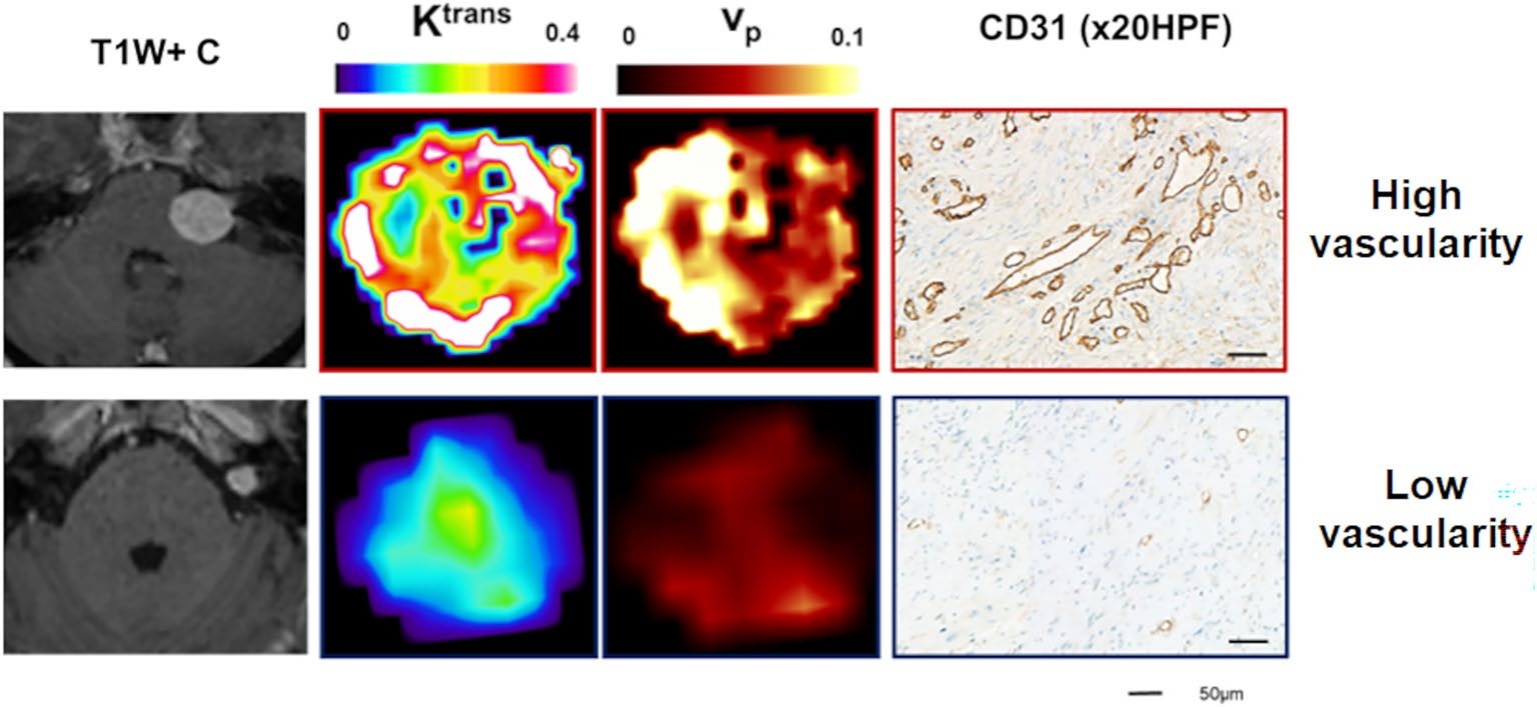
Comparison of derived v_p_ and K^trans^ estimates from the single low-dose DCE-MRI acquisition against tissue-derived vascularity metrics. Representative imaging and histology from a patient with a growing highly vascular VS (top row) and a comparatively less vascular static VS (bottom row) are shown. From left to right: T1W post contrast; parametric *K*^trans^ map; parametric *v*_p_ map; and immunostains (*CD31-brown*) demonstrating the comparatively higher microvessel density within the larger growing VS (immunoperoxidase—× 20 HPF).

**Figure 8:**
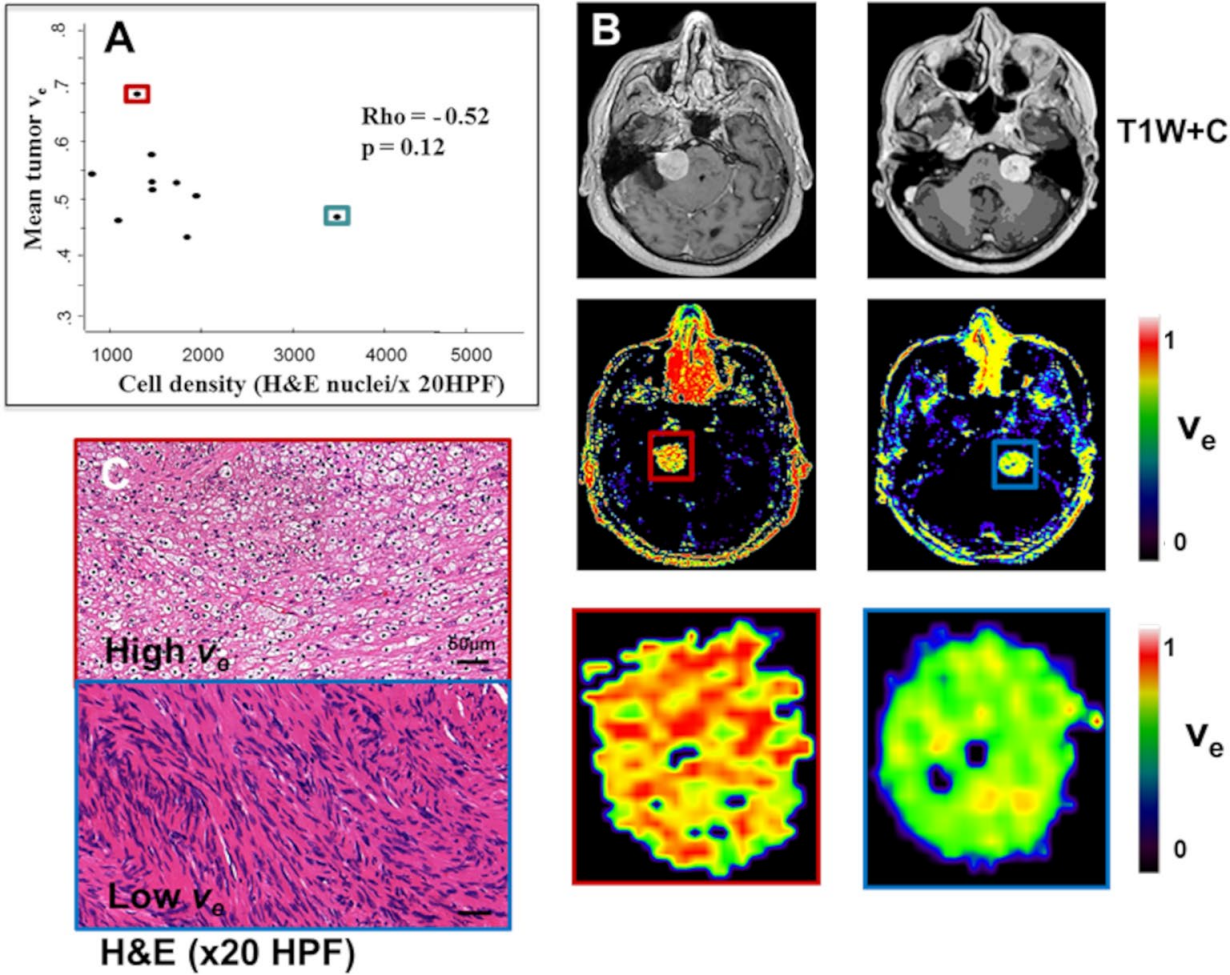
Comparison of derived *v*_e_ estimates from the single low-dose DCE-MRI acquisition against tissue-derived cell density. (**A**) Inter-tumour scatterplot comparison of LEGATOS_LDHS_ derived mean tumour *v*_e_ estimates against H&E cell density (nuclei/× 20 HPF). Spearman’s rho reported. Data from ten tumours shown. (**B**) Representative image sections of a VS with high *v*_e_ estimates (left) and a VS with low *v*_e_ estimates (right). From top: T1W post contrast; parametric *v*_e_ map; and magnification image demonstrating voxelwise heterogeneity in *v*_e_ estimates across the tumour. (**C**) Representative haematoxylin and eosin (H&E, × 20 HPF)–stained sections from the tumour with high *v*_e_ (*top row*) and low *v*_e_ (*bottom row*) respectively. Mean tumour *v*_e_ values for the VS displayed in Panels B and C are outlined in the scatterplot shown in Panel A with a red and blue box, respectively. *HPF* High-powered-field.

Tissue analysis of the 10 resected VSs demonstrated that tissue CD31 microvessel surface area correlated with both *v*_p_ (r = 0.76, p = 0.02) and *K*^trans^ (r = 0.68, p = 0.04) estimates derived using the LEGATOS_LDHS_ approach. Representative imaging and histology from a patient with a growing highly vascular VS (*top row*) and a comparatively less vascular static VS (*bottom row*) are shown in Fig. 7**.** One VS demonstrated disproportionately low mean tumour *v*_p_ compared to mean microvessel SA estimates. Tissue sections of this case demonstrated prominent microcystic change and oedema, and regions of vascular thrombosis likely to attenuate the ‘functional’ vasculature in this tumour (Supplementary Fig. S5). Whilst there was a non-significant inverse correlation between cell density and LEGATOS_LDHS_ derived mean tumour *v*_e_ estimates (rho =− 0.52, p = 0.12), 7/9 of the VSs in this small cohort with a cell density less than 2000 nuclei/ × 20 high-powered-field (HPF) demonstrated a *v*_e_ above 0.5 (Fig. 8), an observation in agreement with the *v*_e_–cell density scatterplot displayed in Fig. 8A**.** Representative imaging from VS with high *v*_e_ (left) and low *v*_e_ (right) are shown in Fig. 8B alongside representative tissue sections from each tumour demonstrating low (Fig. 8C, top) and high cell density (bottom) respectively.

## Discussion

In this study we have demonstrated that through using a newly developed interleaved low-dose DTR DCE-MRI protocol with LEGATOS it is possible to provide accurate, high-spatial kinetic parameter maps following a single low-dose contrast agent injection. Through computer simulations and an in vivo study, we have demonstrated that the LEGATOS-reconstructed dual CNR data from our interleaved high-temporal-high-spatial resolution approach, outperforms use of high-spatial resolution datasets alone, and provides in vivo kinetic parameter estimates comparable to those acquired using a full GBCA dose DCE-MRI acquisition, in keeping with our initial hypotheses.

This is one of the first studies to use a low-dose interleaved acquisition technique containing 3D HT and HS segments for high spatiotemporal resolution brain DCE-MRI. Calagno et al.in a cardiac DCE-MRI study used a full GBCA dose technique, SHILO (Simultaneous HI-/Low-temporal resolution DCE-imaging), which interleaved low spatial resolution single-shot arterial input function (AIF) images with high-spatial/low temporal resolution multi-shot images of the carotid artery vessel wall^21^, in turn providing superior parameter accuracy compared to strategies that sample the AIF and tissue curves at the same temporal rate^21^. Georgiou et al.similarly used interleaved high-temporal and high-spatial resolution segments for quantifying breast tumour blood flow in patients with primary breast cancer undergoing neoadjuvant chemotherapy, demonstrating that treatment related changes in blood flow could be reliably estimated using this interleaved technique^22^. In contrast to the presented work, however, a full-dose of GBCA was used (0.1 mmol/kg) for this interleaved breast DCE-MRI acquisition, and whereas the HT segments were used for tracer kinetic modelling, the HS series were used only qualitatively for the purpose of reporting and breast tumour volume estimation by the radiologist.

Within the brain few studies have investigated use of reduced GBCA dose DCE-MRI^29^. Filice et al., for example, evaluated DCE-MRI in adult patients with glioblastoma using either half dose of high-relaxivity GBCA gadobenate (0.05 mmol/kg) or a full-dose of standard relaxivity GBCA gadoterate (0.1 mmol/kg)^29^, demonstrating that administration of half dose of the high-relaxivity contrast agent gadobenate resulted in better parameter estimation through improving reduced T2*-shortening effects and improved VIF estimation^29^. In this study we have demonstrated the ability to provide accurate, whole-brain high-spatial resolution kinetic parameter estimates from DCE-MRI following a single low-dose GBCA injection of around one quarter of the standard dose. The optimal dose for low-dose high-spatial-resolution DCE-MRI is, however, unknown and for this feasibility study a fixed-volume injection approach was therefore used, adopting a 3 ml bolus volume based on previous DCE-MRI studies from our institution^2–5,18^.

In well enhancing tumours such as VS, K^trans^ estimates derived from the low-dose interleaved DCE MRI protocol were comparable to the full GBCA dose method, and this was the case across the low GBCA dose range for this VS patient cohort (0.016–0.030 mmol/kg). It is, however, possible that in patients with larger body mass or less avidly enhancing tumours such as chordoma this fixed-volume approach was too low, and a larger or weight adjusted GBCA dose could have resulted in more precise kinetic parameter estimation. In highly vascular tumours, such as the VS shown in Fig. 4, underestimation of parameter values by the low dose acquisition was also observed within tumour regions of high K^trans^, vp and v_e_ respectively, and such differences may reflect inaccuracies in parameter estimation due to the lower CNR. Conversely increased T2* and water exchange effects, and the effects of residual contrast agent from the prior low dose injection (even with repeat baseline R1 mapping) may reduce parameter accuracy and precision when using a full GBCA dose^15,30,31^, leading to erroneously high voxelwise parameter values within some tumours. A limitation of the present study is that there were differences in the GBCA concentration time-course length between the low and full dose acquisitions. Whilst such differences could in theory explain some of the differences seen in K^trans^ values between the full dose and low dose acquisition it is unlikely to be a significant contributing factor to parameter accuracy and precision, with both acquisitions being longer than the recommended 5 min in the literature for accurate parameter estimation^32–34^. Larger studies incorporating a wider range of intra- and extra-axial CNS tumours and a variable GBCA dosing regimen (we for example suggest using 0.025 mmol/kg i.e., one quarter of the standard dose as a starting point for further dose optimization) with matched low and full dose protocol lengths should be performed, however, to better understand this new technique’s limitations and establish the optimal GBCA dosing regimen for each tumour type to maximise parameter accuracy and precision.

For studying the microvasculature in skull base tumours T1W DCE-MRI offers distinct advantages over more common dynamic susceptibility contrast based perfusion techniques, through avoidance of susceptibility artefacts and T2^*^ decay^35^. Previous patient studies in VS for example have demonstrated that kinetic parameters derived using dual temporal resolution DCE-MRI not only accurately reflect intertumoural differences in tumour vascularity metrics and tumour macrophage content but also correlate with tumour growth rate and size^3,4,19^. Such parameters have also shown predictive potential in patients undergoing stereotactic radiosurgery^5,36^, and the ability to provide high-spatial resolution interrogation of macrovascular permeability and flow changes during anti-angiogenic treatment^2,37^. The role of DCE-MRI has been less extensively studied in chordoma and chondrosarcoma but previous studies have demonstrated that DCE-MRI derived parameters can be used to differentiate chordoma/ chondrosarcoma from other pathologies such as skull base metastases^38,39^ and spinal giant cell tumours^40^. Crucially in patients undergoing repeated contrast agent exposures the ability to derive high-spatial resolution kinetic parameters following a low-dose injection could have considerable clinical relevance. In clinical trials of antiangiogenic therapies for example, the use of this technique would allow significant reduction in contrast dose, and thereby potential brain gadolinium deposition, whilst supporting more accurate estimates of individual parameters and increased statistical sensitivity to therapy induced changes in individuals or groups.

Whilst there is currently a lack of evidence that gadolinium retention causes disease or disorders in subjects with normal kidney function, this is still an active area of investigation^6,7,9,11,41,42^, and in many other countries regulators recommend to use the ALARA (as low as reasonably achievable) principle with regard to GBCA dose^41,42^. Beyond the use of contrast free techniques and alternative contrast agents with higher relaxivity^41,43,44^, other authors have explored use of deep learning/ machine learning techniques for enhancing the level of contrast of low-dose MRI acquisitions or predicting virtual contrast enhanced images from multiple zero-contrast sequences^41,42,45,46^. Such methods have to date, however, not be applied for dose reduction in DCE-MRI and there are recognised limitations to currently available deep learning/machine learning methods, such as image distortions in virtual or enhanced contrast maps and the presence of low-contrast false-positive enhancements^45^. An evaluation of whether a low-dose GBCA injection can be used for both DCE-MRI and deriving clinically acceptable post contrast structural T1 imaging, was beyond the scope of the current study, but future studies should look to assess application of a low GBCA dose protocol for deriving structural imaging in a range of CNS tumours.

Data from our study suggests that our low-dose technique works best in avidly enhancing tumours such as schwannomas, and our computer simulations support this, demonstrating higher CNR and parameter accuracy with increasing “true” tumour K^trans^ values. Compared to schwannomas the imaged chordoma/ chondrosarcoma cohort in our study demonstrated higher mean SFE using both low-dose and full-dose GBCA acquisitions, and the cause for this is likely multifactorial. Prior to DCE imaging all but one of the chordoma/chondrosarcoma cohort had previously been treated with either prior surgery or radiotherapy and this likely impacted on the vascular integrity and blood supply of these tumours^5^, and may in part explain the observed differences in K^trans^ and v_e_ seen in these tumours relative to VS. Furthermore whereas VS show intense GBCA enhancement^47^, enhancement in chordoma and chondrosarcoma is often heterogenous, with a recognised quarter of such tumours demonstrating no or only mild post GBCA enhancement^48–50^. The pattern of contrast enhancement can be related to the pathological features of these tumours, with chordoma displaying organized lobules of mucinous and gelatinous contents and chondrosarcoma displaying heterogeneous or arabesque like enhancement due to the presence of fibrovascular bundles surrounding cartilaginous nodules^49,50^.

Kinetic parameter estimates derived from low spatial, HT resolution DCE-MRI have been shown in previous in vivo studies to accurately reflect inter-tumour differences in tissue vascularity metrics in sporadic VS and correlate with both tumour growth rate and differences in macrophage content^3,4^. The low spatial resolution in derived parameter maps limited accurate assessment of intratumoural heterogeneity, however. In the present study we demonstrated that high-spatial resolution derived microvascular parameters derived following a low-dose injection can differentiate inter-tumour differences in vascular density and cell density. A limitation of DCE-MRI as well as any non-invasive imaging technique is that the true in vivo tissue perfusion parameters are not known. These parameters can only be extrapolated from structural features detectable on ex vivo tissue specimens such as microvessel surface area and density. Vessel thrombosis and interstitial oedema may attenuate perfusion within a tumour, leading to mismatch between the observed structural and ‘functional’ vasculature, and this may in part explain the discrepancy between imaging and tissue observed in one tumour in this study.

## Conclusion

We have demonstrated that through the combined use of a newly developed interleaved single-injection, low-dose DTR DCE-MRI protocol it is possible to provide tissue-validated, high-spatial kinetic parameter maps following a single low-dose contrast agent injection. We have demonstrated through computer simulations and an in vivo feasibility study that the contrast-to-noise of acquired data from our interleaved DCE approach is sufficient for accurate kinetic parameter estimation and that this low-dose technique provides in vivo kinetic parameter estimates comparable to those acquired using a full GBCA dose DCE-MRI acquisition.

### Supplementary Information

Supplementary Information.

## Data Availability

The datasets generated during and/or analysed during the current study are available from the corresponding author on reasonable request.
