# Supplementary material for: Low-dose GBCA administration for brain tumour dynamic contrast enhanced MRI: a feasibility study
Source: Sci Rep. 2024 Feb 28;14:4905. doi: 10.1038/s41598-024-53871-x (PMC10902320; doi:10.1038/s41598-024-53871-x)

**Supplementary Tables**

**Table S1: Comparative table of tumour DCE-MRI–derived microvascular parameters and scaled fit error (SFE) values when using either full GBCA dose LEGATOS_DICE_ or low GBCA dose LEGATOS_LDHS_ method.**

The mean parameter value (± within group SD) for each tumour group is shown. The p value shown represents the difference between tumour groups. The p value was calculated using 1-way ANOVA with Bonferroni correction.

|  | **Parameter estimate** | **Tumour (N = 20)** | | | |
| --- | --- | --- | --- | --- | --- |
|  |  | **Schwannoma**  N = 14 | **Chordoma**  N = 3 | **Chondrosarcoma**  N = 3 | **P value** |
| **LEGATOS_DICE_  derived parameters**  **(Full GBCA dose acquisition)** | **Mean tumour v_e_ (SD)** | 0.54 ± 0.12 | 0.36 ± 0.16 | 0.58 ± 0.15 | > 0.05 |
|  | **Mean tumour K^trans^ (SD), min^-1^** | 0.17 ± 0.07 | 0.10 ± 0.10 | 0.14 ± 0.07 | > 0.05 |
|  | **Mean tumour v_p_ (SD)** | 0.05 ± 0.02 | 0.05 ± 0.05 | 0.04 ± 0.02 | > 0.05 |
|  | **Mean tumour SFE (SD)** | 0.19 ± 0.06 | 0.44 ± 0.17 | 0.27 ± 0.11 | **0.006** |
|  | **Mean % voxel exclusion (SFE > 0.5) (SD)** | 4.77 ± 6.92 | 34.0 ± 35.8 | 13.7 ± 10.5 | **0.02** |
| **LEGATOS_LDHS_  derived parameters**  **(Low GBCA dose acquisition)** | **Mean tumour v_e_ (SD)** | 0.54 ± 0.08 | 0.23 ± 0.26 | 0.60 ± 0.08 | **0.001** |
|  | **Mean tumour K^trans^ (SD), min^-1^** | 0.16 ± 0.06 | 0.05 ± 0.08 | 0.14 ± 0.05 | > 0.05 |
|  | **Mean tumour v_p_ (SD)** | 0.05 ± 0.02 | 0.04 ± 0.05 | 0.04 ± 0.02 | > 0.05 |
|  | **Mean tumour SFE (SD)** | 0.39 ± 0.07 | 0.73 ± 0.26 | 0.56 ± 0.07 | **0.0003** |
|  | **Mean % voxel exclusion (SFE > 0.5) (SD)** | 20.1 ± 12.8 | 75.1 ± 41.9 | 47.6 ± 15.8 | **0.0007** |

# **Supplementary Figures**

**Supplementary Figure S1: Histograms of the percent deviations (PD, %) of the “measured” parameter values (K^trans^, v_p_, v_e_) from the “true” values, and the scaled fitting error (SFE)**

Histograms shown for three noise levels (NL): 1) NL_HT_, a lower noise level resembling the high temporal resolution (HT) DCE series (top row); 2) NL_Hs_, a higher noise level resembling the high spatial resolution (HS) DCE series (bottom row); and 3) a mixed noise level (NL_HT-HS_) resembling the in vivo HT-HS interleaved DCE data (middle row). Note that the distributions of the PD and SFE at the three different noise levels (NL_HT,_ NL_HS_ and NL_HT-HS_) have Gaussian-like or normal distributions. The mean ± SD of each PD (%) or SFE distribution is shown in the upper left corner of each histogram respectively.

**
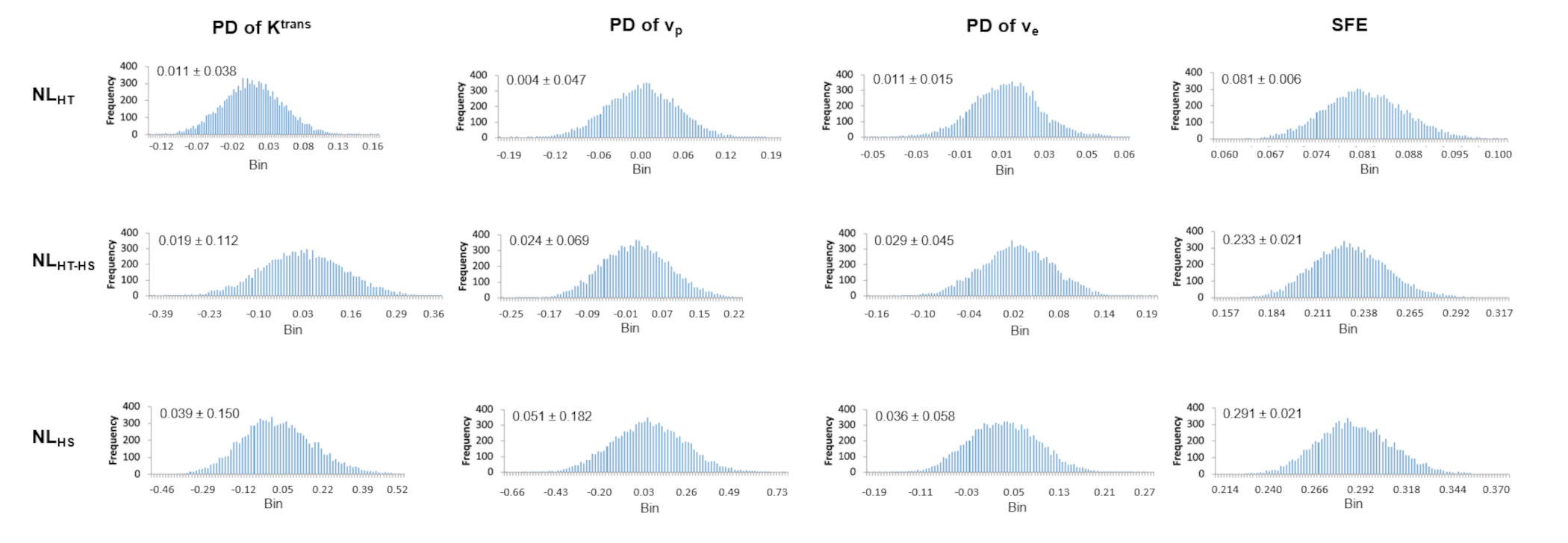
**For each noise condition, a total of 10,000 repetitions were performed and used in each histogram construction (N = 10,000). Each percent deviation bin is expressed as a ratio, (PD/100) within each histogram.

**Supplementary Figure S2: Differences in tumour voxel concentration-time curves ((C_t_(t)) derived from either the low-dose (LDHT-LDHS) or full GBCA dose DCE-MRI acquisition.**

Tissue-concentration curves ((C_t_(t)) derived from a typical tumour VS voxel when using either the low-dose (LDHT-LDHS, *left*) or full GBCA dose (LDHT-FDHS, *right*) DCE-MRI acquisition are shown. Typical tumour VS voxel curves shown for three patients injected with 0.030/0.070 (*top row*), 0.020/0.080 (*middle row*) and 0.016/0.084 (*bottom row)* mmol/kg of GBCA are shown. Whereas the 1^st^ pass of the in vivo acquired C_t_(t) curves from the low-dose and full-dose acquisitions are comparable, the parenchymal segment of C_T_(t)^LDHT-LDHS^ are noisier than the C_T_(t)^LDHT-FDHS^ reflecting the lower GBCA dose. Voxel estimates for scaled fitting error (SFE) and K^trans^ are shown along with the voxelwise calibration ratio (ratio_calib_) for each voxel curve. Note that the length of the LDHT-FDHS concentration-time curve (400 seconds) is shorter than the LDHT-LDHS curve (751 seconds).

**
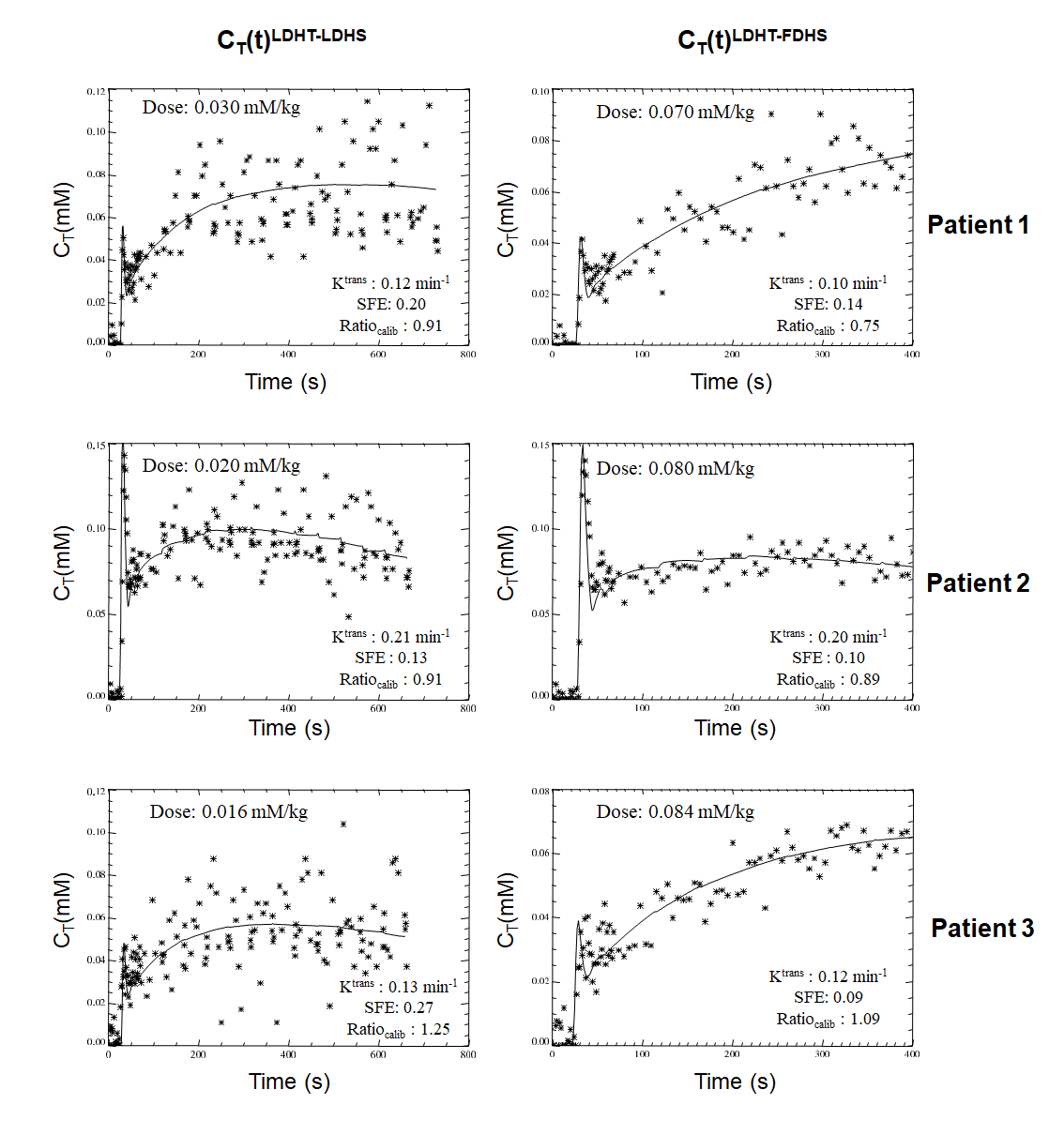
**

**Supplementary Figure S3: Images of CNR, SFE and LEGATOS derived kinetic parameters from a patient with a sporadic VS imaged using both the low-dose *(top)* and full GBCA dose *(bottom)* DCE-MRI acquisitions.**

High-spatial resolution maps (voxel size = 1.0 x 1.0 x 2.5mm) of contrast-to-noise ratio of the washout/parenchymal phase (CNR_HS parenchymal_), SFE and *K*^trans^ from the in vivo acquired low-dose C_T_(t)^LDHT-LDHS^ and full-dose C_T_(t)^LDHT-FDHS^ curves.

Note the comparable *K*^trans^ estimates of the VS derived from the C_T_(t)^LDHT-LDHS^ and C_T_(t)^LDHT-FDHS^ curves despite the five-fold difference in GBCA dose (*short arrows*); mean tumour *K*^trans(LDHT-FDHS)^ = 0.173 s^-1^ and *K*^trans (LDHT-FDHS)^ = 0.175 s^-1^. By comparison, normal appearing brain on the *K*^trans^ map derived from the C_T_(t)^LDHT-LDHS^ appear noisier reflecting the rapid increased uncertainty due to extremely low CNR in these areas (*long arrow*).


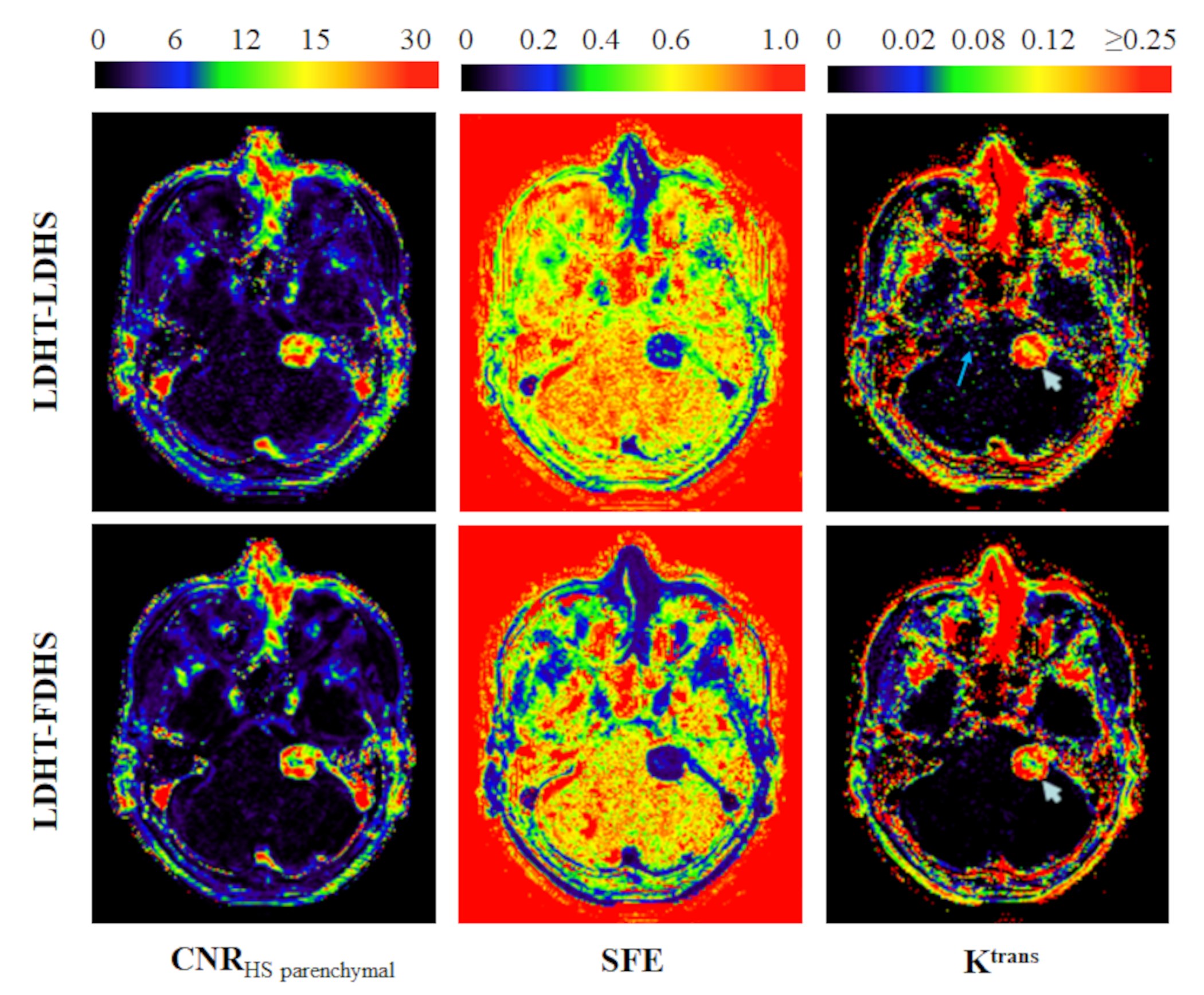
CNR_HS parenchymal_ = mean of parenchymal phase enhancement / standard deviation of baseline frames, calculated from the concatenated HT and HS concentration curves in each voxel of the merged 4D concentration volume.

**Supplementary Figure S4:** **Comparison of tumour mean values of *K*^trans^, *v*_p_ and *v*_e_ obtained using the low-dose LEGATOS_LDHS_ and full -dose LEGATOS_DICE_ methods.**

A: The correlation between mean tumour LEGATOS_LDHS_ and LEGATOS_DICE_ derived estimates are shown. The results are reported using Pearson’s product moment correlation coefficient (*r*). Data from twenty tumours shown.

B: Bland-Altman plots (left column). Bias/mean difference (dotted line) and 95% limit of agreement (LOA, mean difference ± 1.96 · SD) reported on each figure.

**
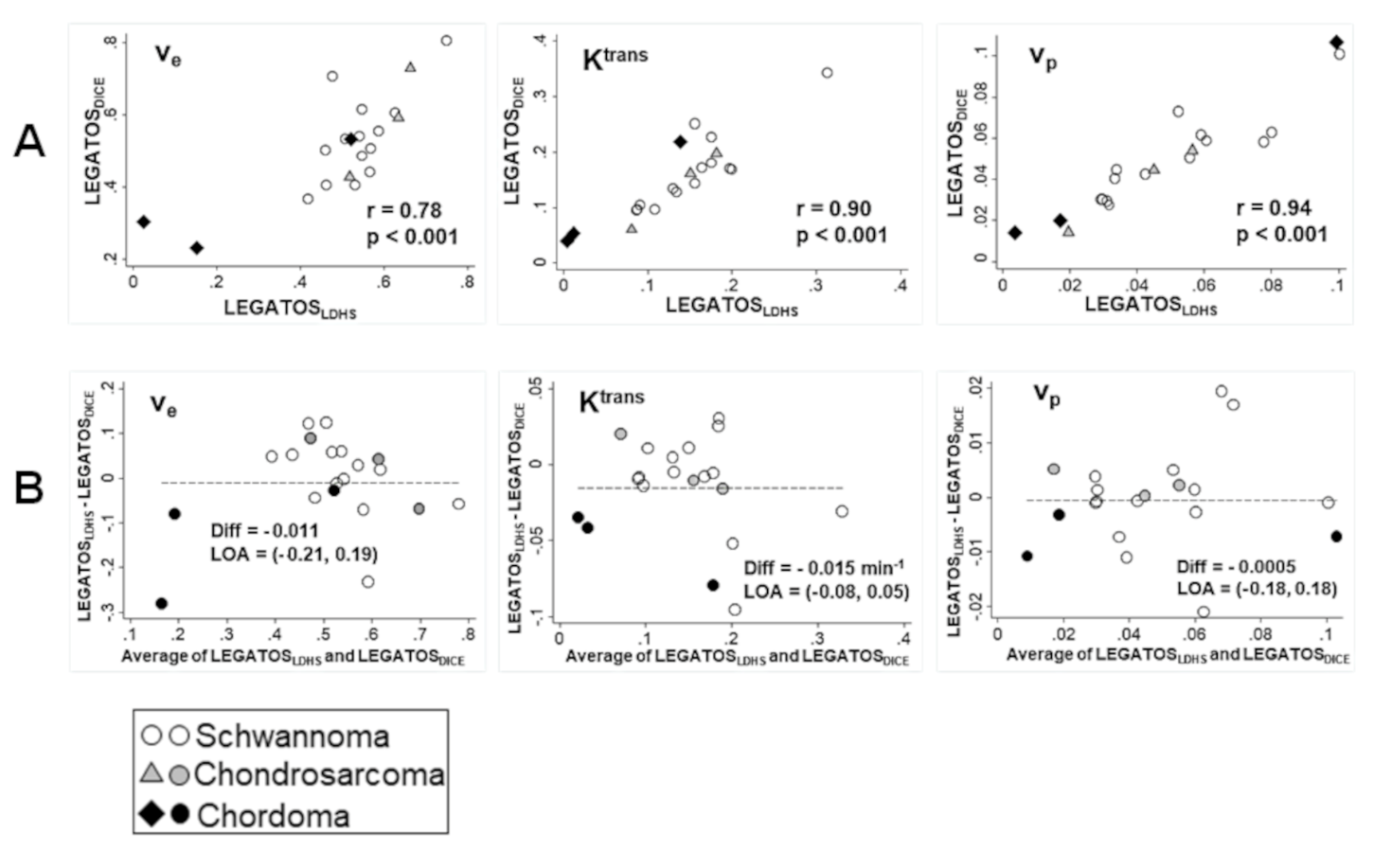
**

**Supplementary Figure S5: Imaging and tissue parameters in a patient with low *v*_p_ and high microvessel density.**

 A: T1W post contrast demonstrating large right sided VS, note the heterogenous enhancement within the tumour.

B: Parameter maps of *v*_e_, *K*^trans^ (min^-1^) and *v*_p_ derived from the single-injection low-dose (*top row*, LEGATOS_LDHS_) and dual injection DTR DCE-MRI datasets (*bottom row*, LEGATOS_DICE_). Note the high *v*_e_ but low *v*_p_ values within the tumour.

C: Haematoxylin and eosin (H&E, x 10) and CD31 immunostains (*CD31-brown*, x10 immunoperoxidase) demonstrating a region of microcystic change/ interstitial tumoural oedema and low microvessel density within the tumour.

D: Haematoxylin and eosin (H&E, x 10) and CD31 immunostains (*CD31-brown*, x10 immunoperoxidase) demonstrating regions of vascular thrombosis (*) within the tumour.


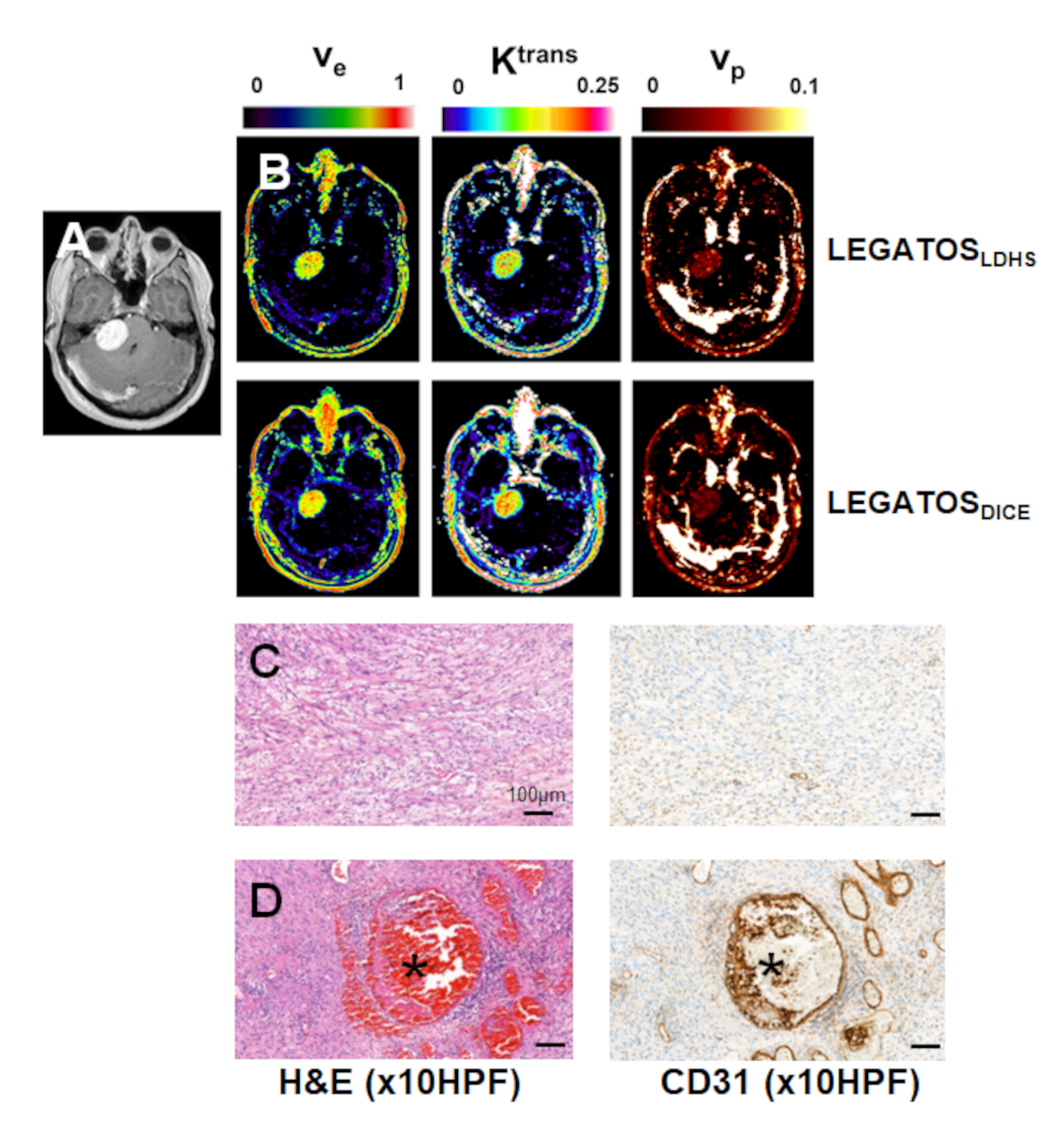

Supplement: Supplementary file 1 — Supplementary Information. [file 41598_2024_53871_MOESM1_ESM.docx]
